# A nurse-led multidisciplinary service for Nipple-Areola complex tattooing after breast cancer: reporting on a complex intervention with TIDieR analysis

**DOI:** 10.1186/s12912-024-02456-0

**Published:** 2024-10-25

**Authors:** Deborah Maselli, Martina Torreggiani, Monica Guberti

**Affiliations:** 1 Azienda USL - IRCCS of Reggio Emilia, Reggio Emilia, Italy; 2 University of Modena and Reggio Emilia, Modena, Italy

**Keywords:** Medical tattoing, Women health services, Breast cancer nursing, Complex intervention, Oncology nursing, Health service research

## Abstract

**Background:**

The Nipple-Areola Complex (NAC) tattooing can restore physical and mental integrity after breast cancer, but it is not always easily accessible for women. This paper aims to report on the development of a multidisciplinary nurse-led service for NAC tattooing for women who underwent breast cancer surgery with NAC removal to allow its thorough review and replication.

**Methods:**

The Medical Research Council’s framework for developing complex healthcare interventions was followed. According to the results of a literature review, and the context analysis, an initial intervention was planned. The Template for Intervention Description and Replication checklist was chosen to ensure the quality and completeness of the intervention description.

**Results:**

The Breast Unit and the Research departments were engaged; three nurse-tattooists were selected; the informative material was created and shared with patients, families and local associations, involving them actively. Finally, the setting and the materials were defined. A monthly schedule of activities was set: patients with the indication for NAC tattooing were contacted by the nurse case manager. Each treatment involves 3–4 sessions, 30–40 days apart, in an ambulatory setting. It consists of NAC shaping and tattooing with a dermographer and sterile needles.

**Conclusion:**

Implementing freely and equally multidisciplinary nurse-led clinics might provide this treatment ensuring the patient’s quality of life and nurse competence. The NAC tattooing is a complex intervention that represents the final part of the breast cancer surgical care pathway.

**Supplementary Information:**

The online version contains supplementary material available at 10.1186/s12912-024-02456-0.

## Background

Breast cancer is the most frequently diagnosed malignant tumor in women [1]. When surgical treatment is indicated, it can affect quality of life even years later [2]. It is well known that many patients experience difficulties related to the complexity of reconstructive treatment with the removal of the Nipple-Areola Complex (NAC) [2, 3]. The loss of harmony in the body image’s perception can compromise identity and relationships with others [4, 5]. Providing a multidisciplinary nurse-led service for NAC tattooing as the final part of the breast oncological clinical pathway might improve women’s physical and psychological rehabilitation and wellness. The NAC dermopigmentation reconstructs the NAC appearance [6]. International evidence suggests that dermopigmentation is a satisfactory and well tolerated nonsurgical technique, with low complications, costs, and waiting times [7, 8]. The tattoo can be performed alone or with other reconstruction techniques [9–11], under local anesthesia. Moreover, it represents the only possible option in case of some contraindications, such as damaged tissues, comorbidities, anxious states related to past hospital experiences [8, 12]. Initially introduced by Rees [13] and recently refined [14, 15], the steril/semi-sterile technique is performed by a specifically trained professional through the introduction of bioabsorbable pigments into the superficial papillary dermis with a disposable needle, through a demographer or manually [16]. The lack of in-clinic NAC tattooing services is a barrier to equal access to this type of care. In many countries, this technique is usually performed by professional tattoo artists [17], with considerable costs for patients. Literature shows variability in professional and management aspects according to healthcare contexts [17, 18]. The training and the competence involved in tattooing are variable and poorly described in literature; nevertheless, the nurse appears to be the professional most frequently involved [18]. Establishing the appropriate competence and the replicability of NAC tattooing in-clinic services represents a challenge for research, as also understand the relationship with quality-of-life outcomes and assess the feasibility of nurse-led services [18]. The NAC tattooing is an example of a complex intervention in healthcare: following the definition, the complexity of an intervention is determined by its properties and by the number of components, structures, and roles involved, as well as the expertise and skills required [19]. Identifying the context of applicability and the appropriate outcomes and assessment processes is essential. To our knowledge, studies have yet to apply this framework in developing and evaluating such interventions. The purpose of this paper is to report on the development of a multidisciplinary nurse-led service for NAC tattooing for women who underwent breast cancer surgery with NAC removal to allow its thorough review and replication.

## Methods

**Fig. 1:**
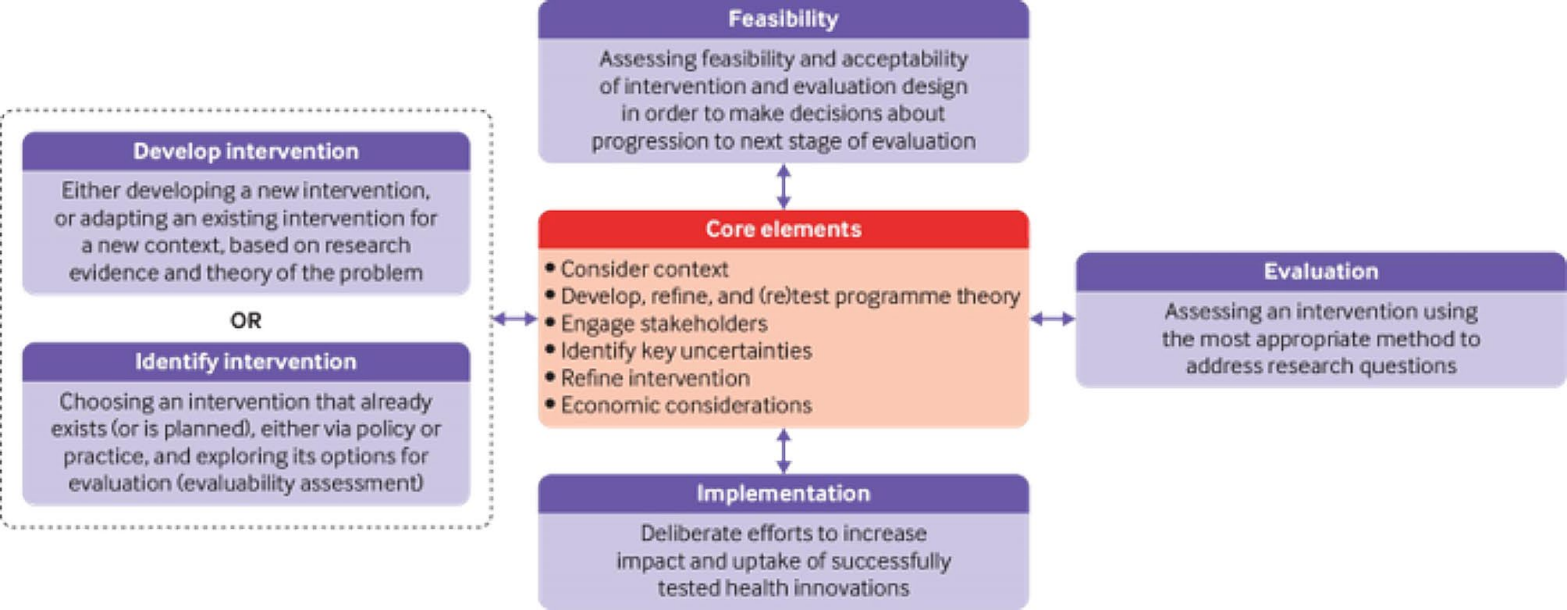
- MRC framework for complex interventions by Craig et al. (2008) [20]

The framework of the Medical Research Council (MRC) for developing and implementing complex interventions in healthcare was chosen to achieve the objective, specifically the first of the following phases [19]: (1) development or identification of the intervention; (2) feasibility; (3) evaluation; (4) implementation. These methodological steps will be crucial in answering our research question: how can an NAC tattooing service be developed and adapted to the local healthcare context? The other phases will be assessed in future studies. This flexible model (Fig. 1) permits beginning research projects at any point and revising previous phases and elements if uncertainties remain unresolved. Some core elements are shared: context analysis, developing and refining program theory, engaging stakeholders, identifying key uncertainties, refining the intervention, and economic considerations. The setting of the study is Santa Maria Nuova Hospital of Azienda USL - IRCCS of Reggio Emilia.

As summarized in Table 1, we transferred the phases into Work-Packages (WPs). The WP1 is the object of this report and presents the first development phase. It is composed of three steps:

- Step A: this step aims to examine the evidence available about the NAC tattooing reconstruction after breast cancer surgery: the grounding literature exploration was synthetized with a scoping review.
- Step B: it aims to identify theories, context elements, and part of interests in developing the intervention. The context examination was performed by sharing moments with key stakeholders, such as patients, associations, families, and professionals. Patient and public involvement are crucial to collecting information about the implementation context of a co-designed project and promoting participation through meetings and events. Identifying professionals, healthcare services, materials, and settings was planned.
- Step C: this phase aims to develop an initial plan of activities by integrating collected evidence (step A) and context analysis (step B). The following elements were identified: activities scheduling, waiting list management, materials storage, ordering and tracking, personnel management, documentation and procedure development, and collection of photographic/audio-video materials.

**Table 1:**
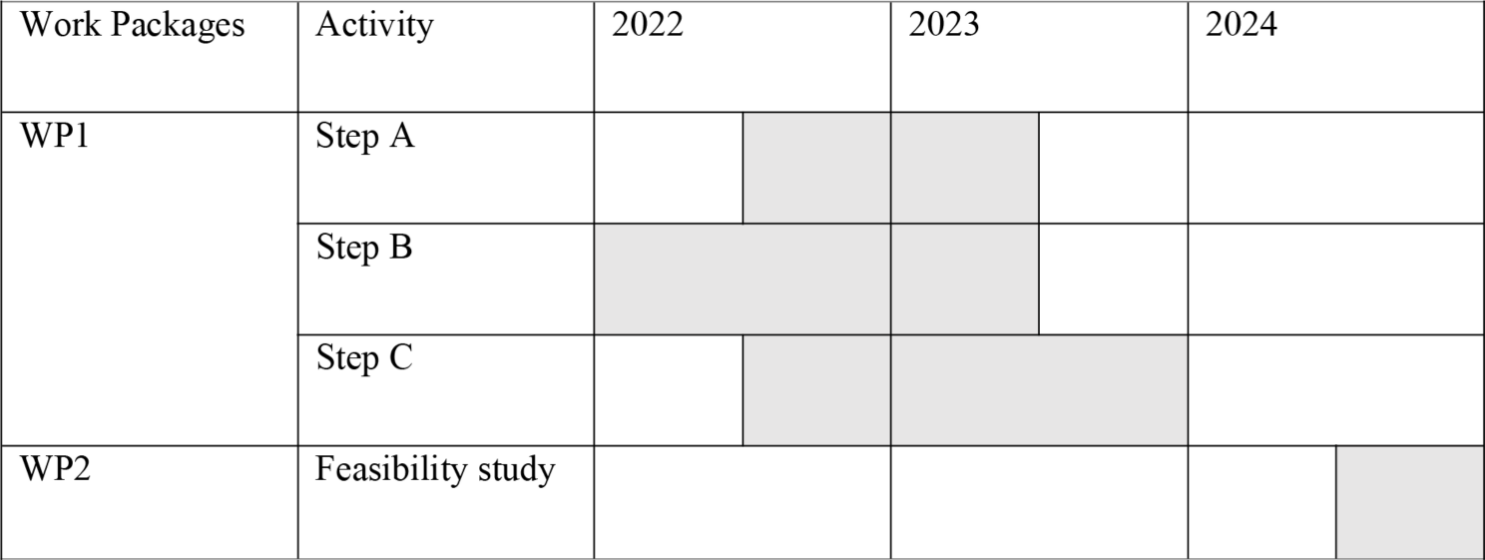
Work packages and timeline of phase 1 and phase 2 of the study methodology

Moreover, the Template for Intervention Description and Replication (TIDieR) will describe the intervention in detail to ensure the quality and completeness of the description. This validated tool is needed as it helps capture the accurate composition and interacting features of a complex intervention with reliability [21]. At each stage of the process, all activities were continuously monitored and refined.

The WP2 will be started in a further study, and it consists in the feasibility study: it aims to test the planned intervention by assessing the prelaminar organizational feasibility, with an initial focus on safety, overall satisfaction, and economic evaluation. The following will be the detected indicators: N completed treatments, N adverse events, N activity days, N tattoos performed, timing/duration of the tattoo session, and cost evaluation.

### Findings

This report describes the activities conducted in the development phase of the ARCADE service.

#### WP1 - step A: evidence about NAC tattooing

The scoping review results [18] confirm that NAC tattooing is a safe and satisfactory intervention that can finally restore the integrity of appearance to women who underwent demolitive cancer treatments, improving their quality of life with promising cost-effectiveness. The paper points out that professional training on tattooing techniques is variable and poorly described: nurses were the most involved, followed by medical staff and tattooists. The endpoints explored in the literature focused mainly on the satisfaction rate of the aesthetic result. Finally, the most frequent limitations were poor evaluation criteria and the use of study-specific questionnaires based on previous work. The studies described some experiences of nurse-led services. These findings present a relevant field of research and allow us to orient resources and plans for a local intervention development.

#### WP1 - step B: the context analysis

As the context can determine the success of a new service introduction, we focused on these actions:

##### Understanding the local regulatory framework

In the Italian universalistic healthcare system, the tattooing for medical purposes is performed on people who need to cover pathological skin conditions, restore the appearance of healthy skin, or as an adjunct to reconstructive surgeries [16]. In this case, it can be a phase of the medical procedure that intervenes downstream of the diagnostic-therapeutic course to restore a balance and recover, psychologically, the patient’s well-being and an excellent final aesthetic outcome, according to the complete concept of health: a full condition of life from the individual, economic and social point of view. The medical tattoo of NAC is the only one legislatively regulated [22] and included in the Essential Levels of Care (and therefore supported by the National Health Service).

##### Identification of health services and professionals

We worked on building a solid core competence integrated within the services: the Research & EBP Unit of the Health Professions Department, the Scientific Direction of the Cancer Research Institute, and the Integrated Breast Unit were involved for each level of responsability. Three motivated and trained nurses were chosen to lead the service with the collaboration and supervision of the breast unit medical staff through an internal public selection. The dermopigmentation will be performed mainly by one highly specialized nurse with extensive experience in women’s oncological surgery. The scientific and management aspects of the research were handled by the Research & EBP Unit.

##### Patient and public involvement

We created a brochure that provides patients with necessary information about the treatment. We presented the project to patients, families, citizens, and local associations. In 2022, we organized a competition among residents to propose the name of the new clinic, with a large and enthusiastic participation. One hundred forty-two name proposals with motivations were collected, all dense of meanings evoking concepts of renaissance and harmony comeback. The winner’s name was ARCADE: the word reminds the service activities (“Ambulatorio Ricostruzione Capezzolo Areola DErmopigmentazione). The name is also similar to “*Arcadia*” a famous region celebrated in Greek mythology as an unspoiled place of harmonious wilderness. Stakeholders like patient associations were essential in raising awareness and spreading evidence-based information among the community.

#### WP1 - step C: the planning of activities

Combining scientific evidence with local considerations, we started the development of an initial intervention. As each treatment involves about 3–4 sessions 30–40 days apart, we proposed that the clinic activities would be done monthly, with an initial patient group meeting to introduce the treatment and clarify any doubts. Regular briefings and debriefings were planned to share procedures, management aspects, and any issues that would have arisen. The following logistical and technical aspects were defined and shared:

- *Population*: the intervention is dedicated to adult women who underwent mastectomy with NAC removal within six months, with medical indication to the NAC dermopigmentation.
- *Setting*: the clinic activities will be carried out in ambulatory, day-case settings in Santa Maria Nuova Hospital of Reggio Emilia.
- *Contact*: elegible patients would be contacted individually by the breast nurse case manager to agree on the date and time of the session.
- *Documentation*: instruments for tracking and ordering materials, waiting list management, and clinical documentation were prepared.

It was expected that for each of the first sessions, it would be possible to treat patients who need one bilateral and one unilateral tattoo or up to three unilateral ones. The procedure of the intervention with timing and materials information (Annex A) was shared.

##### The planned NAC tattooing intervention

The intervention is described with the TIDieR Checklist in the Annex B. The technical procedure and training are based on the best scientific evidence and valid and well-established referrals in this field [23]. Thanks to a local cream anesthetic, the treatment is painless and odorless. The tattooing with natural pigments does not interfere with instrumental examinations or breast implant replacement. The bioresorbable pigments made the color naturally and gradually fade (in the days following the session, about 30–40%). Natural pigments differ from artistic pigments precisely because of their reabsorption properties in the body over time, which also allow, thanks to periodic sessions, to give conformity to the changes brought about by skin aging. The dermopigmentation can also be performed on any previous scars in the areola-nipple area by including them in the tattoo design. The reproduction of the areola-nipple complex will be done with a 3D (light-dark) effect technique by inserting bioresorbable pigments into the epidermis with disposable needles. The instrument used to carry out this technique is the dermograph. This comprises a central body, which allows the needle oscillation speed adjustment, a handpiece, and sterile disposable needles. Considering that the third session best fixes the color, an annual appointment for a touch-up will be necessary. In the first dermopigmentation session, the nipple-areola design (color, shape, and overall appearance of the areola) is created. During the 30 min necessary for the anesthetic cream to work, an individual interview is conducted to clarify all aspects of the procedure. The tattoo session lasts, on average, one hour. It has been pointed out that no preparation is necessary before the session, only a few precautions to be followed after the treatment: upon its completion, the treated area should be cared as any wound. Nurses will leave an oily gauze in place to be removed about 3 h later. At home, patients should perform regular hygiene of the new areola with mild soap and water, without rubbing but dabbing it. Applying an emollient cream for 3 to 4 days, 1 or 2 times a day, and as needed is recommended. In the following days, small scabs may form, which should be left in place until their natural fall, which will occur approximately after 10 to 15 days; this expedient is essential to prevent local infections and to allow the pigment to fix itself at its best. During this period, it is necessary to avoid swimming pools, saunas, steam baths, intense sports activities, laces, synthetics, sun exposure, and parfum use.

## Discussion

This paper reports on the development of a multidisciplinary nurse-led service that provides NAC tattooing reconstruction after cancer surgery. The chosen methodology was appropriate, as it permitted us to understand which factors determined the success or the failure of ARCADE in the local context. Evidence-based healthcare is known to be a cycle process informed by the best available evidence, the context in which care is delivered, the individual patient, and expertise of the health professional [24]. However, where evidence is sparse, more inclusive, and less traditional, methods can allocate value and relevance to findings, as they can inform healthcare decisions and resolve many important evaluation questions [19]. Hence, we have seen that the tailoring to the specific context is essential to address a real health need appropriately. This experience promoted multidisciplinary health research on innovative care models and services in oncology, focusing on cancer survivors’ quality of life and economic aspects. The project has a potential positive impact on professional competence, patients’ quality of life, and organization. The selection process of the nurse tattooists was completed assuming that the expertise of health professionals is the key to guarantee the quality and feasibility of the intervention. Being a professional in the field of medical dermopigmentation means not only knowing the techniques of color design and use but also having a broad and in-depth view of dermatologic, oncologic, and surgical elements [25]. Healthcare workers can promptly recognize and treat potential complications, like infections or skin necrosis [26]. In addition to the technical aspect, they should focus on the therapeutic relationship established by elaborating on body image and identity after cancer, as included in a referral team on decision support and territorial services. Nursing competence in this field should also include patient counseling about body image, scar management, identity role issues after cancer, decision support on postmastectomy options, and quality of life [27]. The definition of a skills’ profile, the competence maintaining, and the specialized professional support network (e.g., dermatology, radiology) will be the elements of future research refinement.

We will care about providing fulfilling information and safety along all the activities, collecting feedback, and monitoring complications. As suggested by previous literature [28], the intervention’s impact on patients may vary from cosmetic outcomes to satisfaction and quality of life: that brings strong significance to implementing services that restore psycho-physical integrity. The following steps will provide accuracy in the quality assessment of the intervention and will focus on systematically evaluating the quality of life aspects implicated in NAC tattooing. The preliminary phases of WP1 are continuously implicated in this evaluation: as we have seen, the context is determinant: the stakeholder feedback and the resource evaluation may be decisive in the success or failure of an intervention. This will be monitored in further evaluations, and the following elements will be defined: tools for intervention prescription and accountability, structured cost-effectiveness evaluation, and staff management. This research presents some limitations: first, the results are not generalizable, as they refer to the context of application; second, they are to be considered preliminary and referred to activities description and processing. Despite the considerations above, this project on breast cancer care and survivorship highlights some strengths, exploring with reliable methodology fields of advanced care nursing in leading new quality-of-life services in oncology care that is valuable and worthy of exploration. To our knowledge, this is the first report that used the TIDieR checklist to describe a NAC tattooing service as a complex intervention, a fundamental step to ensure transparency and replicability in health service research. More structured specialist training pathways are necessary to better define competencies, quality levels and appropriateness, across medical tattooists’ professionals. As also supported by recent literature on the topic [29], further research on psychological benefits of medical tattooing among breast cancer survivors is strongly necessary to promote its integration in the cancer care. Studies highlighting the multiple positive impacts will be helpful: professionals might benefit from this experience in growing competence, satisfaction, motivation, engagement with continuous care, and teamwork. Finally, the organization will benefit from this implementation, improving health outcomes affordably and cost-effectively.

## Conclusion

Providing a NAC tattooing service after breast cancer is an intervention that closes the circle of the oncological care pathway. Implementing these services has an extensive meaning for women’s global health, promoting organizational efficiency, safety, and nurses’ professional value. A valid methodological referral is essential to assess a complex intervention’s development and piloting. The next steps are required to systematically evaluate the quality of life issues implicated in NAC tattooing and the sustainability of this multidisciplinary nurse-led service.

## Electronic supplementary material

Below is the link to the electronic supplementary material.

Supplementary Material 1

Supplementary Material 2

## Data Availability

No datasets were generated or analysed during the current study.

## Abbreviations

NAC: Nipple- Areola Complex
Azienda USL - IRCCS: Azienda Unità Sanitaria Locale - Istituto di Ricovero e Cura a Carattere Scientifico
WP: Work-Packages
TIDieR: Template for Intervention Description and Replication
ARCADE: Ambulatorio Ricostruzione Capezzolo Areola DErmopigmentazione
