# Supplementary material for: A nurse-led multidisciplinary service for Nipple-Areola complex tattooing after breast cancer: reporting on a complex intervention with TIDieR analysis
Source: BMC Nurs. 2024 Oct 25;23:785. doi: 10.1186/s12912-024-02456-0 (PMC11515310; doi:10.1186/s12912-024-02456-0)
Supplement: Supplementary file 1 — Supplementary Material 1 [file 12912_2024_2456_MOESM1_ESM.docx]

Annex B – Description of the intervention with the TIDieR (Template for Intervention Description and Replication) Checklist.

| **Brief Name** | **Why** | **What Materials** | **What** **Procedures** | **Who Provided** | **How** | **Where** | **When and How Much** | **Tailoring** | **Modifications** | **How Well** |
| --- | --- | --- | --- | --- | --- | --- | --- | --- | --- | --- |
| Nipple-Areola Complex Tattooing | When surgical treatment for breast cancer is indicated, body image issues can affect the quality of life, identity and relationships.  The dermopigmentation is performed on people who need to cover pathological skin conditions, restore the appearance of healthy skin, or as an adjunct to reconstructive surgeries. | Local anesthetic cream; steril gauzes, towels, and gloves; bioresorbables pigments; dermograph and desposable steril needles. Information material like brochures, consent and anamnesis sheet; computer programs to manage list and appointments; camera. | In the first dermopgmentation session, the nipple-areola design is created (color, shape and overall appearance of the areola). During the minutes required for the anesthetic, the nurse conducts an individual interview to clarify all aspects of the procedure. The nurse will trace the material and any adverse effects during treatment. The nurses will leave an oily gauze on the spot to be removed about 3 hours later. At the end of the session, the patient will be asked to document the tattoo project with photographs from NAC. At home, patients should perform normal hygiene of the new areola with water and mild soap, without rubbing, but dabbing it. It is recommended to apply an emollient cream for 3 or 4 days, 1 or 2 times a day and as needed. | The breast surgeon recommends the treatment and supervise patients; the case manager nurse contacts women and manages waiting lists; the trained nurse tattooists perform tattoos and follow-up care. The research unit assesses and monitors the sessions, outcomes and other scientific and management aspects. | The appointment will be arranged by telephone, as also any follow-up contact. The sessions will be carried out in person. | Hospital Surgical Ambulatorial setting. | After six month from the last surgery, but still after the indication of the surgeon. A session can last 60 min for a single NAC, 90 min or more for bilateral dermopgmentation.  Three sessions are scheduled at a distance of 30-40 days. One-year follow-up tattoo is planned. | Timing, personalization of shape and color, other technical aspects are considered. Patients have personal stories and different needs: the result will be customized and adapted to the characteristics and will of the patient. In some cases a fourth session is necessary. | Modifications may follow the monitoring initial results of the patient (feedback on satsfaction, adverse reactions, adhesion and cancellations), new requests and calendar of the agenda.  It will be possible to modify the operating modalities of the treatments in order to face the necessary resources and costs (the continuous formation of professionals, materials, working hours and other events). | Adherence to treatment will be evaluated by keeping track of the procedure completed by each individual patient. Checklists on sessions will also be evaluated. |
