# Supplementary material for: A nurse-led multidisciplinary service for Nipple-Areola complex tattooing after breast cancer: reporting on a complex intervention with TIDieR analysis
Source: BMC Nurs. 2024 Oct 25;23:785. doi: 10.1186/s12912-024-02456-0 (PMC11515310; doi:10.1186/s12912-024-02456-0)
Supplement: Supplementary file 2 — Supplementary Material 2 [file 12912_2024_2456_MOESM2_ESM.docx]

**Annex A – Procedure, timining and materials**

| **Activity** | **Materials** | **Time** |
| --- | --- | --- |
| Welcoming and data collection  Informed consents | Sheets and informative materials | 10 |
| Photo prior the treatment | Camera | 2 |
| NAC Project (color and shape)   - With reconstructed NAC - Without reconstructed NAC - Without reconstructed bilateral | Pigments, dermographic pen, transparent film | 10  20  30 |
| Anesthetic cream application | Anesthetic cream | 30 |
| Tattooing   - Monolateral - Bilateral | Steril gauzes, towels, gloves and sleeve; bioresorbables pigments; dermograph and desposable steril needles. | 30  60 |
| Photo of the result | Camera | 2 |
| Oily gauze application | Oily gauze | 2 |
